# Phytochemical characterization of the *Vochysia rufa (Vochysiaceae)* extract and its effects on oxidative stress in the pancreata of streptozotocin-induced diabetic rats

**DOI:** 10.1371/journal.pone.0184807

**Published:** 2017-10-05

**Authors:** Neire M. de Gouveia, Wellington F. Rodrigues, Raquel M. F. de Sousa, Luciana K. Calábria, Antonio V. Mundim, Camila B. Miguel, Carlo J. F Oliveira, Javier E. Lazo-Chica, Alberto de Oliveira, João H. G. Lago, Vagner B. dos Santos, Claudimir L. do Lago, Foued S. Espindola

**Affiliations:** 1 Instituto de Genética e Bioquímica, Universidade Federal de Uberlândia, Uberlândia, Minas Gerais, Brazil; 2 Departamento de Ciências Biofuncionais, Faculdade Morgana Potrich, Mineiros, Goiânia, Brazil; 3 Curso de Pós-graduação em Ciências da Saúde, Universidade Federal do Triângulo Mineiro, Uberaba, Minas Gerais, Brazil; 4 Instituto de Química, Universidade Federal de Uberlândia, Uberlândia, Minas Gerais, Brazil; 5 Faculdade de Ciências Integradas do Pontal, Universidade Federal de Uberlândia, Ituiutaba, Minas Gerais, Brazil; 6 Faculdade de Medicina Veterinária, Universidade Federal de Uberlândia, Uberlândia, Minas Gerais, Brazil; 7 Laboratório Morfofuncional, Centro Universitário de Mineiros, UNIFIMES, Mineiros, Goiânia, Brazil; 8 Instituto de Ciências Biológicas e Naturais, Universidade Federal do Triângulo Mineiro, Uberaba, Minas Gerais, Brazil; 9 Centro de Ciências Naturais e Humanas da Universidade Federal do ABC, Santo André, São Paulo, Brazil; 10 Instituto de Química, Universidade de São Paulo, São Paulo, São Paulo, Brazil; Stellenbosch University, SOUTH AFRICA

## Abstract

Aqueous extract of macerated *Vochysia rufa* stem bark has been commonly used in the treatment of diabetes. Therefore, we evaluated the antihyperglycemic and antioxidant effects of an extract of *V*. *rufa* on the pancreata of streptozotocin (STZ)-induced diabetic rats. Animals received one of the following treatments daily by oral gavage: water (diabetic-control), *V*. *rufa* extract (diabetic-*V*. *rufa*), or glibenclamide (diabetic-GBD). Total antioxidant capacity; levels of thiobarbituric acid reactive substances, reduced glutathione, and sulfhydryls; and superoxide dismutase, catalase, and glutathione peroxidase (GPx) activities were measured in the pancreas. Biochemical analysis of serum total cholesterol and fractions, triglycerides, creatinine, urea, acid uric, ALP, γ-GT, AST, and ALT was performed, and pancreatic β-cells positive for insulin were evaluated by immunohistochemistry. Rats treated with extract exhibited a decrease in fasting blood glucose compared with levels in diabetic control rats. GPx activity and sulfhydryl levels were significantly lower in diabetic-*V*. *rufa* rats compared with those of diabetic-control rats. *V*. *rufa* extract acted to normalize the biochemical alterations found in diabetic rats (diabetic-controls), as demonstrated by increases in urea, HDL, ALP, AST, and ALT. Reduction in blood glucose was independent of an increase in insulin. The *V*. *rufa* extract was found to be composed of free sugars (inositol, galactose, glucose, mannose, sucrose, arabinose, and ribose) as the main metabolites. Thus, aqueous extract of the stem bark of *V*. *rufa* is capable of reducing blood glucose, resulting in an antioxidant effect on the pancreatic tissue of STZ-diabetic rats.

## Introduction

Diabetes mellitus (DM) is a metabolic disease that affects more than 347 million people worldwide [1] and is characterized by high blood glucose due to inadequate insulin production or insulin activity. Previous works have demonstrated that persistent hyperglycemia in patients with diabetes induces glucose autoxidation and protein glycosylation and that these processes increase the production of free radicals including reactive oxygen species (ROS) [2, 3]. ROS concentrations are modulated by antioxidant enzymes such as superoxide dismutase (SOD), catalase (CAT), and glutathione peroxidase (GPx), in addition to non-enzymatic scavengers [4]. Increased activity of the three primary scavenging enzymes has been demonstrated in various tissues from diabetic animals [5].

In recent years, traditional and alternative medicines have been investigated for their utility in the treatment of diabetes. Studies have suggested that certain botanical polysaccharides isolated from *Ophiopogon japonicus* [6], *Lycium barbarum* [7, 8], *Opuntia dillenii* [9], and *Achillea santolina* [10] exhibit hypoglycemic activity. In the case of *A*. *santolina*, this hypoglycemic activity is attributed to the presence of compounds with antioxidant properties and free radical scavengers in this plant [10]. Approximately 100 species of *Vochysia* (Vochysiaceae), including large trees and shrubs, occur throughout tropical America from Mexico to Peru. Several of these species have been used by traditional Amerindian communities in South America for a variety of therapeutic purposes linked to inflammation, including treating skin sores and relieving respiratory ailments such as asthma and pulmonary congestion [11]. The methanolic extract of the leaves of *Vochysia tucanorum* exhibits antiulcer activity due to the presence of gastric pentacyclic triterpenes, such as betulinic acid, epibetulinic acid, and erythrodiol, as well as mixtures and derivatives of ursolic and oleanolic acid [12]. The antibacterial activity of sericic acid, the main active constituent isolated from the extract of *Vochysia divergens* bark, may explain and justify the popular use of this plant in treating infectious diseases [13]. Extract of the bark of *Vochysia rufa* contains phenolic compounds, coumarins, anthraquinone heterosides, triterpenoids, and saponins [14]. Moreover, our previous work has demonstrated that *V*. *rufa* reduces oxidative stress in the liver [15]. Taken together, these findings suggest that studies to evaluate the effects of *Vochysia* compounds, including those in *V*. *rufa*, should be continued.

*V*. *rufa*, popularly known as “quina-doce”, has been used in folk medicine to treat type 1 and type 2 diabetes mellitus in the state of Minas Gerais, Brazil; however, its antidiabetic and antioxidant effects and phytochemical profile have not yet been elucidated. Thus, we evaluated the efficacy of treatment with an extract of *V*. *rufa* stem bark in STZ-treated diabetic rats as well as its potential antihyperglycemic and pancreatic antioxidant effects.

## Materials and methods

### Collection of *V*. *rufa* and preparation of the extract

Stem barks of *V*. *rufa* Mart. were collected in the Cerrado biome accessible to the Federal University of Uberlândia in the outskirts of Abadia dos Dourados/MG, Brazil (latitude 18° 27′ 50.5″, longitude 47° 23′ 37.2″) during the months of October–February, 2010–2011. The plant material was botanically identified at the Institute of Biology of the Federal University of Uberlandia, and deposited in the University Herbarium (HUFU) with voucher specimen number 58,888.

*V*. *rufa* Mart. stem barks were dried in an oven at 35°C and then powdered in an electric mill. The powder (200 g) was extracted with distilled water by maceration for 24 h (1:5 w/v). The extract was filtered and centrifuged at 4,400×*g* for 15 min at 4°C. The supernatant was frozen at –20°C and then lyophilized for a yield of approximately 6% (w/w). The crude extract was stored at –20°C until analysis.

### Identification of sugars by capillary electrophoresis—Tandem mass spectrometry

Analyses were carried out in a capillary electrophoresis–mass spectrometry (CE-MS) system Agilent 7100 (capillary electrophoresis) interfaced to a 6430 triple-quad mass spectrometer (Agilent Technologies, Santa Clara, CA, USA). The background electrolyte (BGE) was 0.5 mol∙L^-1^ triethylamine (pH 12). Sheath liquid for the electrospray ionization (ESI) source was prepared by diluting the BGE 160 times with 10/90 methanol/water (v/v) and used at a flow rate of 6 μL∙min^-1^. During the electrophoretic run at 25 kV, a backpressure of -20 mbar was applied to the inlet vial for compensation of the ESI suction effect (Do Lago, 2014A). Nitrogen was employed as a nebulizer gas (10 psi) and drying gas (3 L∙min^-1^ at 150°C). Inlet capillary voltage and dwell time were set to 4.5 kV and 200 ms, respectively. Experiments were carried out using a 65-cm long, 50-μm i.d., 360-μm o.d. fused-silica capillary (Agilent Technologies, Redmond, OR, USA). The capillary was preconditioned by washing with 1.0 mol∙L^-1^ NaOH solution (3 min), deionized water (5 min), and BGE (5 min). A thermally isolated case (Do Lago, 2014B) was used to control the temperature of the capillary at 20°C during the electrophoretic run. Samples were hydrodynamically injected at 100 mbar for 10 s. The mass spectrometer was operated in negative-mode selected ion monitoring (SIM) to detect the [M-H]^-^ ions at *m/z* 149 (xylose and arabinose), 163 (fucose), 179 (glucose, fructose, galactose, inositol), and 341 (sucrose) with fragmentor voltage and cell accelerator voltage set at 40 V and 3 V, respectively.

A solution of 3.1 mg∙mL^-1^ crude extract was prepared with deionized water and filtered through a 0.45-μm Millipore filter before use (Millipore, Billerica, MA, USA). Stock solutions (50 mmol∙L^-1^) of arabinose, ribose, fucose, glucose, galactose, inositol, sucrose, xylose, fructose, maltose, and mannose were prepared in deionized water. These sugars were identified by CE-MS of the sample solution after spiking. Quantification with the addition standard method was carried out using a mix containing each sugar at 2.5 mmol∙L^-1^, which was mixed 1:1 (v/v) with the crude extract solution.

### Animals

Male Wistar rats (approximately 8 weeks and 200–220 g) were used for animal experiments and maintained under standard conditions (22 ± 1°C, 60 ± 5% humidity, and 12 h light/12 h dark cycle). Animals were fed a commercial pellet diet (65.82% carbohydrate, 5.36% fiber, 21.0% protein, and 4.96% fat) (BioBase, SC, Brazil) and received water *ad libitum*. For euthanasia, rats were anesthetized with a mixture of cloridrate of ketamine (5 mg/100 g of weight) and cloridrate of xylazine (2 mg/100 g of weight) followed by exsanguination through cardiac puncture and cervical dislocation. All procedures for the handling, use, and euthanasia of the animals were approved by the Ethics Committee in Animal Research of the Federal University of Uberlandia, Brazil (CEUA/UFU 060/10).

### Acute toxicity study

In the acute toxicity study, rats were assigned to one of eight groups (n = 6 animals in each group); each group was administered a different concentration of the aqueous extract of *V*. *rufa* (50, 100, 250, 500, 1,000, 3,000, or 5,000 mg/kg body weight) in a single dose by oral gavage. The control group received water. Animals were weighed at the beginning of the experiment. For the remainder of the study, we chose to use a concentration of 500 mg/kg of aqueous extract of *V*. *rufa* because this concentration has been demonstrated to have important bioactive properties in hepatic tissues of diabetic rats [15] and was found to be non-toxic.

Animals were fasted with water *ad libitum* for 12 h before receiving the extract. Thirty minutes after administration, animals were given free access to food and were observed at intervals of 5 min, 15 min, 30 min, 60 min, 3 h, and 6 h. Animals were assessed twice a day for 14 days in the evening (at 16:00 and 18:00). The weight of each animal was also measured during treatment. We observed the following parameters: changes in skin, hair, and eyes; presence of tremors; muscle tone; changes in salivation, defecation, and urination; lethargy; sleep; arousal; muscle twitching; convulsions; and death of the animals. Animals found to be moribund were euthanized with a mixture of cloridrate of ketamine and cloridrate of xylazine followed by cervical dislocation and were excluded from the experiment.

### Induction of diabetes mellitus and monitoring of body weight and glycemia

Rats were allowed to acclimate to their environment for one week and were subsequently fasted for 24 h. Rats were then intraperitoneally anesthetized with xylazine/ketamine (1:1, v/v) followed by intraperitoneal injection of 40 mg/kg STZ (Sigma-Aldrich Corporation, St. Louis, MO, USA). STZ was freshly dissolved in 0.01 M citrate buffer at pH 4.5, and the injection volume was 2 mL/kg. Animals were fasted for another 90 min after the injection. Ten days after the STZ injection, rats with a fasting blood glucose level greater than 250 mg/dL were used for subsequent experiments [16, 17]. Body weight and blood glucose levels were monitored in animals undergoing the different treatments (water, glibenclamide, and *V*. *rufa*) at three different time points: the beginning of the experiment, after 21, and after 43 days. Blood was collected from the tip of the tail vein, and the fasting blood glucose level (after 6 hours of fasting) was measured using reactive strips for blood glucose (Contour Glucose Test Strip, Bayer, Mishawaka, IN, USA).

### Experimental design

Animals were assigned to one of six groups of five animals each: (i) the negative-control group, which received 1 mL of distilled water for 43 days; (ii) the *V*. *rufa* group, which animals received 500 mg/kg *V*. *rufa* extract for 43 days; (iii) the glibenclamide (GBD) group, which received 6 mg/kg GBD (Biosintética Farmacêutica Ltda, Brazil) for 43 days; (iv) the diabetic-control group (STZ^+^), which was treated with STZ and received 1 mL of distilled water for 43 days; (v) the diabetic-*V*. *rufa* group, which was treated with STZ and received 500 mg/kg *V*. *rufa* extract for 43 days; and (vi) the diabetic-GBD group, which was treated with STZ and received 6 mg/kg of GBD for 43 days).

The extract was diluted in distilled water and administered in the afternoon by oral gavage for 43 days. At the end of the experiment, animals were fasted for 12 h after anesthesia, and blood samples were collected from the hepatic portal vein. Pancreata were dissected immediately, washed in saline (0.9% NaCl), frozen in liquid nitrogen, and stored at -80°C prior to biochemical analyses and analysis of biomarkers of oxidative stress. Other portions of the pancreas were washed in saline and immediately fixed in 10% buffered formalin. All experiments with animals were repeated twice.

### Serum biochemical measurements

Total cholesterol, triglyceride, urea, creatinine, alkaline phosphatase (ALP), gamma-glutamyltransferase (γ-GT), aspartate amino transferase (AST), alanine amino transferase (ALT), HDL cholesterol (HDL-C), and uric acid levels were determined from serum samples. All parameters were measured in a Clinical Analysis Laboratory at the Faculty of Veterinary Medicine of the Federal University of Uberlandia with a ChemWell Automated Analyzer (Awareness Technology Inc., Palm City, FL, USA) via colorimetric methods using commercial kits (Labtest Diagnostica, Brazil).

### Tissue preparation

Four pancreata from each group were homogenized on ice in 10 volumes of homogenization buffer [20 mM Tris-HCl (pH 7.4), 2 mM dithiothreitol, 1 mM benzamidine, 0.5 mM phenylmethanesulfonyl fluoride, 0.5 mM aprotinin, and 0.1 mM Pefabloc). The homogenate was centrifuged at 10,000×*g* for 5 min at 4°C. Protein concentrations were measured by the Bradford method [18].

### Biomarkers of oxidative stress in the pancreas

Total antioxidant capacity (FRAP) was evaluated using the test described by Benzie and Strain [19]. In this test, the reduction of Fe^3+^ 2,4,6-tri(2-pyridyl)-s-triazine (TPTZ) complex (colorless) to Fe^2+^-tripyridyltriazine (blue), formed by the action of electron-donating antioxidants at low pH, is monitored by measuring the change in absorbance at 593 nm and recorded against a reagent blank after a 30-min incubation at 37°C. Lipid peroxidation in pancreatic tissue was estimated colorimetrically as described by Hermes-Lima et al. [20]. This method measures the levels of thiobarbituric acid-reactive substances (TBARS). The samples were homogenized in 1.1% phosphoric acid (1:10, w:v) and then mixed with 1% TBA/50 mM NaOH/0.1 mM BHT solution and 7% phosphoric acid. Subsequently, samples were heated for 15 min at 100°C, and then 1.5 mL butanol was added. Finally, tubes were vigorously vortexed and centrifuged for 5 min in a benchtop centrifuge. The organic layer was removed and placed in cuvettes; absorbances at 532 and 600 nm were measured. Results are expressed in nmol∙g^-1^ of tissue. Sulfhydryl levels were determined using 5,5-dithiobis(2-nitrobenzoic acid) (DTNB), as described by Faure and Lafond [21]. The glutathione S-transferase activity was assayed spectrophotometrically by measuring the conjugation of GSH to the standard GST substrates 1-chloro-2,4-dinitrobenzene (CDNB) and 1,2-dichloro-4-nitrobenzene (DCNB) according to methods described by Habig et al. [22]. Reductions in absorbance were measured at 340 nm. The readings were observed with a Microplate Reader (VersaMax, Molecular Devices, Sunnyvale, CA, USA).

### Activity of antioxidant enzymes

CAT activity was determined by monitoring the decomposition of hydrogen peroxide at 240 nm, as described by Aebi et al. [23]. The activity of SOD was determined by inhibition of adrenochrome during the oxidation of adrenaline according to methods described by Misra and Fridovich [24]. Results are expressed as units of SOD/μg of protein. The activity of GPx was quantified by oxidation of NADPH in the presence of glutathione reductase (GR), which was measured at 340 nm and is expressed in μmol∙min^-1^∙g^-1^, following the recommendations of Flohe and Gunzler [25]. The amount of reduced glutathione (GSH) was determined as described by Beutler et al. [26] in combination with DTNB, based on the fact that reaction of glutathione with DTNB forms a yellow-colored thiol (TNB).

### Immunohistochemical analysis

For histological processing, pancreata were removed, planed, and fixed on filter paper in formaldehyde for 24 h, followed by storage in 70% alcohol until further use. Pancreata were subjected to processing via dehydration, inclusion, and diaphanization followed by microtomy (Histotechnical PT 05 automatic processor). Blocks were cut to obtain 5-μm sections. Sections were placed on slides (10 slides per pancreas), and the procedure was repeated until all slides contained two sections each. This procedure was performed 10 times without discarding any slices. Slides were pre-treated with 3-aminopropyltriethoxy-silane (Sigma-Aldrich). Sections in glass slides were immersed in xylene for 10 min to eliminate paraffin, dehydrated in absolute alcohol, and re-hydrated with Tris-buffered saline (TBS). Sections were rinsed in TBS and immersed in a 3% hydrogen peroxide–methanol solution for 30 min to block endogenous peroxidase activity, followed by a 30-min incubation at 90°C. Immunolabeling of insulin was performed with a 1:500 dilution of rabbit anti-insulin (AB128018, ABCAM Company Ltd., UK); slides were incubated with this antibody for 2 h at 37°C and subsequently rinsed three times with TBS for 3 min per wash. Next, slides were incubated with peroxidase-conjugated protein A (1:100) for 1 h at room temperature (25°C). Slides were washed again and treated with 3,3-diaminobenzidine tetrahydrochloride (DAB Chromogen Kit, Biocare Medical, Concord, USA) for imaging. Slides were subsequently counterstained with Mayer's hematoxylin and mounted. Images were obtained using a light microscope plus camera (Eclipse 50i, Nikon, Tokyo, Japan) at a magnification of 40×. Evaluation was performed using ImageJ software (http://rsb.info.nih.gov/ij/). Thereafter, we obtained the ratio of the number of stained cells to the area analyzed. The area analyzed was standardized by insertion of a square (250 × 250 μm), which was randomly distributed among the islets to reach a total area of 3.125 mm^2^ per animal. Area was obtained in a mean of 10 islets.

### Statistical analysis

Statistical analysis was performed using the Prism software program (GraphPad Inc., San Diego, CA, USA). Normality (Kolmogorov–Smirnov test) and homogeneity of variance tests (Bartlett's test or F-test) were applied to all variables. Parametric tests (unpaired *t*-test for two groups or one-way analysis of variance with Tukey's multiple comparison post-test for three or more groups) were used for cases with normal distributions and homogeneous variances, and results are expressed as means ± standard errors of the mean (SEM). Non-parametric tests (Kruskal–Wallis test with Dunn's multiple comparison) were used for cases with non-Gaussian distributions, and results are expressed as median, maximum, and minimum values. Differences with p-values < 0.05 (5%) were considered significant.

## Results

### Phytochemical screening of *V*. *rufa*

**Fig 1:**
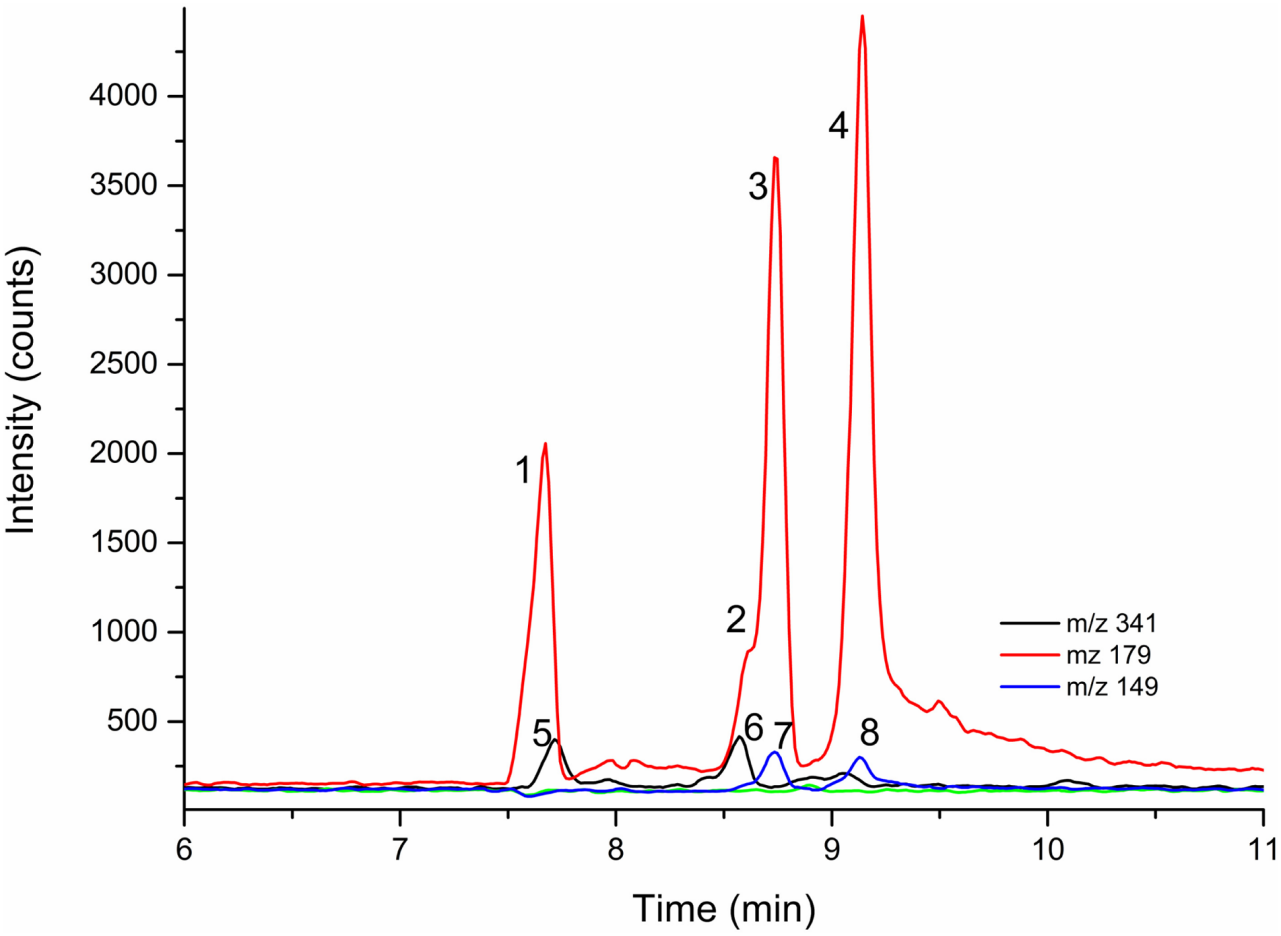
Typical electropherogram of *V*. *rufa* crude extract. (A) Electropherogram and (B) CE-MS spectrum of the *V*. *rufa* crude extract (obtained as described in the Materials and methods). The hexose sugars inositol (1), galactose (2), glucose (3), and fructose (4) were detected at *m/z* 179. Sucrose (5) and another disaccharide (6) were detected at *m/z* 341. Arabinose (7) and xylose (8) were detected at *m/z* 149.

After creating an aqueous extract of *V*. *rufa*, we conducted a phytochemical screening of the extract. The high pH of the BGE allowed the deprotonation of sugars with ionization degrees ranging from 20–62%, and consequently, their electrophoretic separation. Identification of analytes was performed using both migration time and *m/z* of the [M-H]^-^ ion. Electropherograms of the crude extract suggest that it was mainly composed of free sugars, as shown in Fig 1. Four hexoses were simultaneously detected at *m/z* 179: inositol (0.12 mg∙mL^-1^), galactose (0.065 mg∙mL^-1^), glucose (0.47 mg∙mL^-1^), and fructose (0.60 mg∙mL^-1^). Xylose (0.013 mg∙mL^-1^) and arabinose (0.018 mg∙mL^-1^) (*m/z* 149), as well as sucrose (0.22 mg∙mL^-1^) (*m/z* 341) were detected. Fucose (*m/z* 163) was not detected. A possible disaccharide isomer for sucrose with *m/z* 341 was also detected at low concentration.

### Acute toxicity study

The *V*. *rufa* extract did not produce any signs or symptoms of toxicity or mortality at doses of up to 5,000 mg/kg. In addition, markers of hepatic (AST and ALT) and renal (urea and creatinine) function demonstrated that the compound had no adverse systemic effects in these animals. There were no significant increases in ALT, urea, or creatinine levels in the *V*. *rufa* group compared to levels in the negative-control group. While there was a statistically significant increase in AST in the *V*. *rufa* group compared to that in the negative-control group (p<0.05), this increase was less than twofold. In addition, the diabetic-*V*. *rufa* group presented the lowest mean AST concentration among the diabetic groups (Table 1).

**Table 1:** Total cholesterol, triglyceride, creatinine, urea, alkaline phosphatase (ALP), _γ_-GT, aspartate amino transferase (AST), and alanine amino transferase (ALT) levels of nondiabetic (ND) and diabetic (D) rats after daily administration of water, 500 mg/kg *Vochysia rufa* extract (V), or 6 mg/kg glibenclamide (G) for 43 days (n = 5 animals per group, two independent replicates).

Parameter	Groups						
	Negative-control	*V*. *rufa*	GBD	Diabetic-control	Diabetic-*V*. *rufa*	Diabetic-GBD	p-value
Cholesterol (mg/dL)	45.90 ± 2.65	47.24 ± 3.58	38.35 ± 2.26	55.62 ± 5.36	57.52 ± 4.69	47.93 ± 3.31	>0.05
Triglycerides (mg/dL)	84.36 ± 18.75	66.69 ±8.06	70.07 ± 6.83	130.05 ± 17.56	108.10 ± 17.08	114.20 ± 17.11	>0.05
HDL-C (mg/dL)	15.27 ± 2.15	18.84 ± 0.66	16.26 ± 1.10	23.05 ± 0.87*	20.14 ± 0.87	22.81 ± 1.75	<0.05
VLDL (mg/dL)	16.87 ± 3.75	13.34 ± 1.61	14.01 ± 1.37	26.11 ± 3.51	21.62 ± 3.41	22.84 ± 3.42	>0.05
LDL (mg/dL)	13.76 ± 2.10	15.06 ± 3.81	8.08 ± 2.90	6.46 ± 3.70*	15.76 ± 5.89	6.87 ± 3.14	<0.05
Creatinine (mg/dL)	0.63 ± 0.04	0.57 ± 0.04	0.67 ± 0.045	0.55 ± 0.052	0.59 ± 0.04	0.60 ± 0.05	>0.05
Urea (mg/dL)	28.29 ± 2.34	36.76 ± 2.98	40.47 ± 4.48	74.08 ± 6.32*	77.00 ± 3.65	72.69 ± 4.86	<0.05
Uric acid (mg/dL)	2.83 ± 0.95	1.07 ± 0.18	0.88 ± 0.16	1.67 ± 0.80	1.69 ± 0.62	5.38 ± 0.99***	<0.05
ALP (U/L)	219.70 ± 20.21	196.00 ± 19.39	200.80 ± 19.66	1329.00 ± 167.10*	902.70 ± 127.90	1038.00 ± 159.80	<0.05
Ƴ-GT (U/L)	10.64 ± 2.66	12.48 ± 3.48	10.30 ± 2.45	27.71 ± 9.91	32.00 ± 15.03	25.08 ± 11.82	>0.05
AST (U/L)	72.10 ± 12.04	112.80 ± 4.77**	107.40 ± 9.51**	541.00 ± 108.30*	300.00 ± 68.76	416.00 ± 97.61	<0.05
ALT (U/L)	75.20 ± 9.40	91.60 ± 3.58	88.90 ± 5.93	395.00 ± 70.68*	226.70 ± 30.69	330.00 ± 71.93	<0.05

Data are expressed as mean ± SE (n = 10).

*p < 0.05, diabetic-control vs. negative-control;

**p < 0.05 vs. negative-control;

***p < 0.05 vs. diabetic-control.

### Effect of *V*. *rufa* extract on blood glucose and body weight

**Fig 2:**
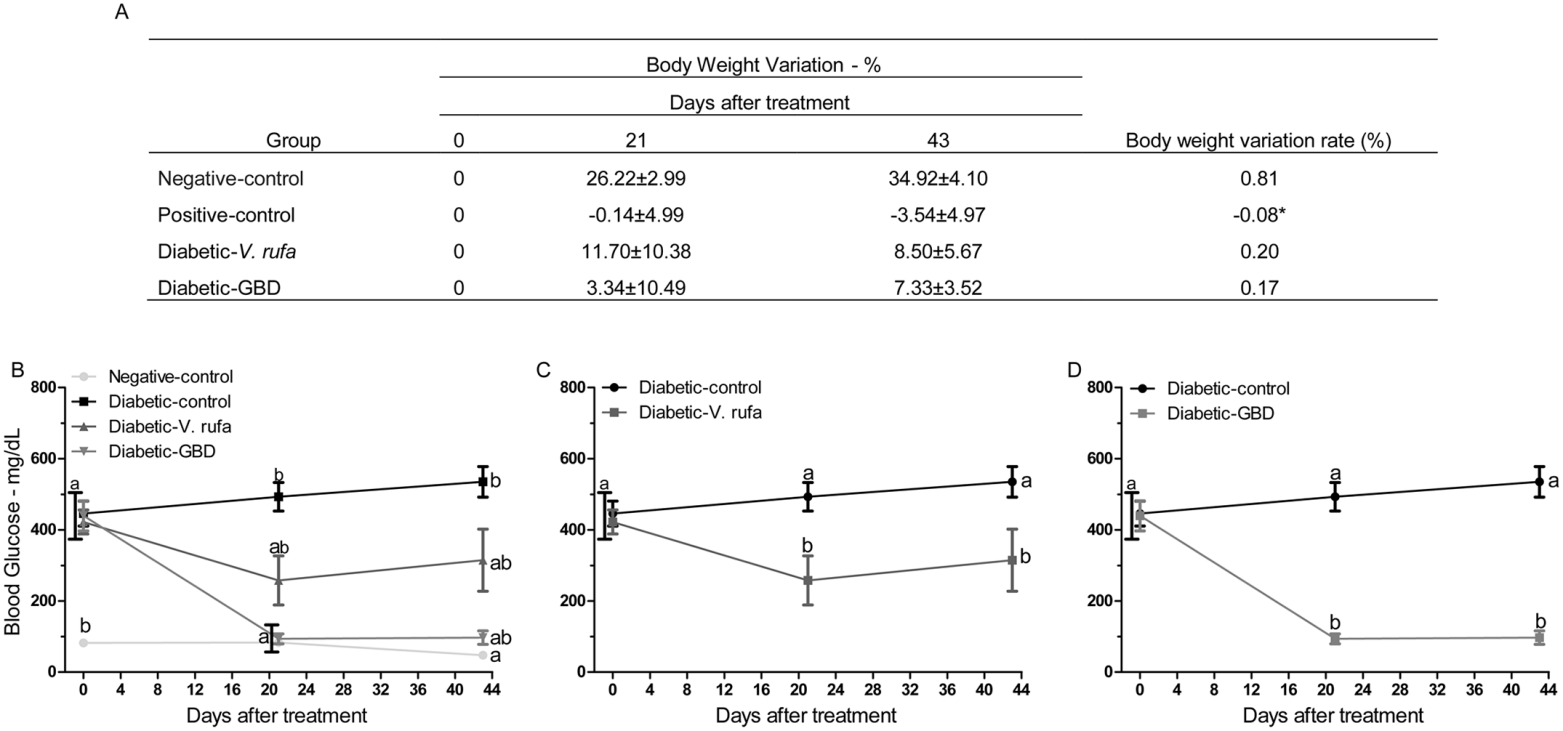
Evaluation of variation in body weight and blood glucose concentrations of experimental groups. Body weight and blood glucose concentrations were measured initially and after 21 and 43 days of daily treatment (n = 5 animals per group, two independent replicates). Rats were divided into negative-control and diabetic groups and were then treated with *V*. *rufa* extract (500 mg/kg) or glibenclamide (6 mg/kg). (A) Rate of variation (Δ) in body weight, which was obtained by dividing the mean of Δ of the body weight in the percentage by Δ of the days of experiment. *p < 0.05. (B) Blood glucose concentrations, which were obtained and compared among different groups. Lowercase letters indicate statistically significant differences between values (*p* < 0.05).

The blood glucose levels and body weights of diabetic animals that received the *V*. *rufa* extract (500 mg/kg) for 43 days are listed in Fig 2. There was a progressive and significant increase in the body-weight variation of negative-control rats, whereas diabetic-control rats demonstrated a decrease in body-weight variation (Fig 2A). A significant increase in blood glucose levels was observed in diabetic-control rats compared with negative-control rats (Fig 2B). After both 21 and 43 days, the blood glucose levels of diabetic-*V*. *rufa* rats were significantly (*p* < 0.05) lower than those of rats in the diabetic control group (Fig 2C). Blood glucose levels in the diabetic-GBD rats also decreased significantly after treatment (Fig 2D).

### Effect of *V*. *rufa* extract on biochemical parameters

*V*. *rufa* and GBD rats demonstrated increased levels of AST (*p* < 0.05) compared with those of negative-control rats. The diabetic-*V*. *rufa* group exhibited no alterations in biochemical parameters compared with the diabetic-control group, whereas the diabetic-GBD group exhibited increased uric acid levels (*p* < 0.05) compared to those of the diabetic-control group (Table 1).

### Effect of *V*. *rufa* extract on biomarkers of oxidative stress in the pancreas

**Fig 3:**
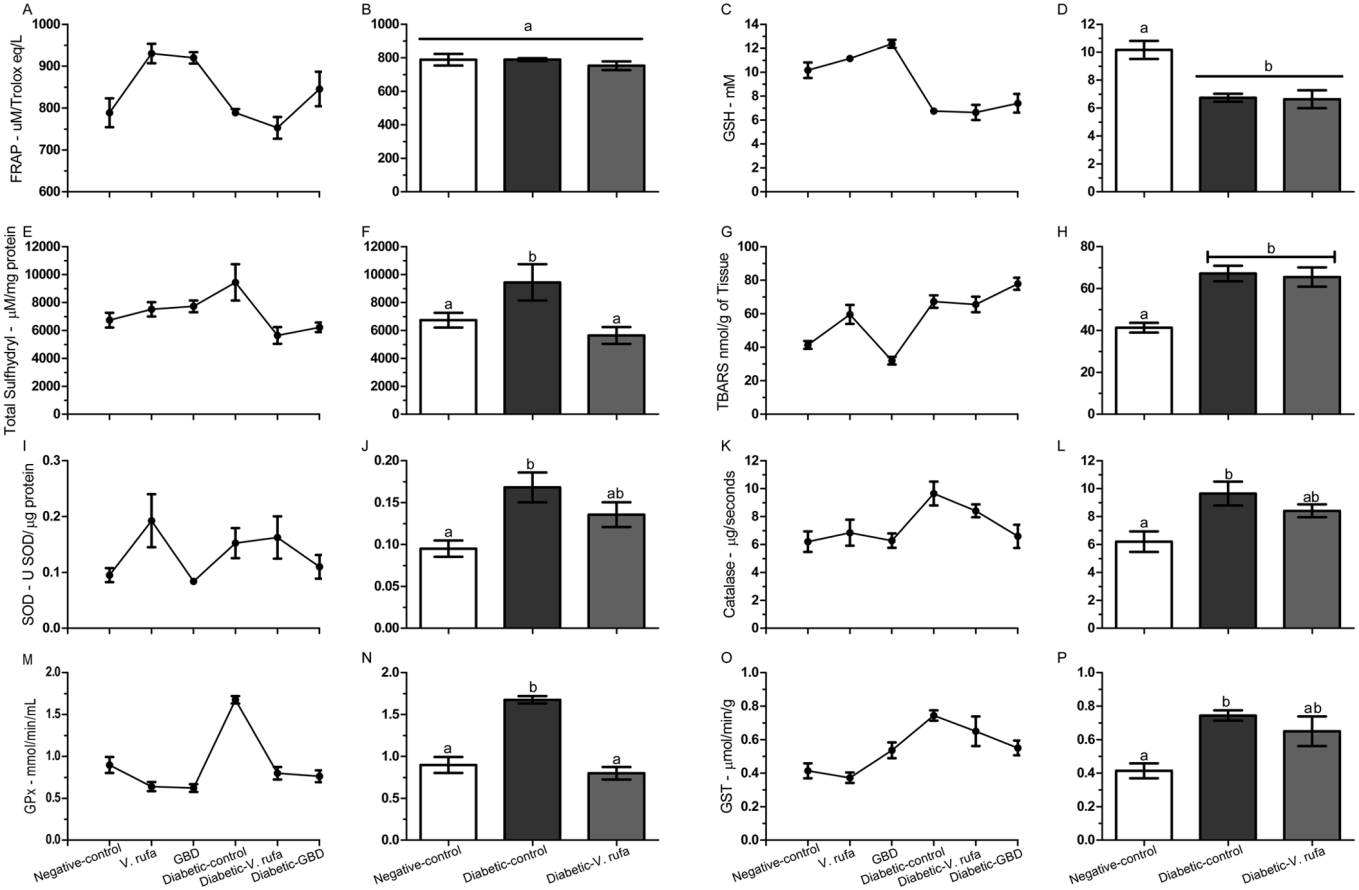
Effects of *V*. *rufa* extract on FRAP, GSH, sulfhydryl, TBARS, SOD, CAT, GPx, and GST levels in the pancreata of nondiabetic and diabetic rats. Evaluation was performed after 43 days of treatment. Rats were divided into negative-control and diabetic-control groups and were then treated with *V*. *rufa* extract (500 mg/kg) or glibenclamide (6 mg/kg) (n = 5 animals per group, two independent replicates). Results for FRAP are presented in μM/Trolox eq/L (A and B), GSH in mM (C and D), sulfhydryl in μM/mg protein (E and F), TBARS in nmol∙g^-1^ of tissue (G and H), SOD in U SOD/μg protein (I and J), CAT in μg/seconds (K and L), GPx in mmol/min/mL (M and N), and GST in μmol/min/g (O and P). Lowercase letters indicate statistically significant differences between values (*p* < 0.05).

Effects of *V*. *rufa* extract on FRAP, GSH, TBARS, and sulfhydryl are shown in (Fig 3A–3H). In the diabetic-control group, we observed increased sulfhydryl and TBARS levels (Fig 3H, *p* < 0.05) in addition to decreased GSH levels (Fig 3D, *p* < 0.05). Diabetic-V *rufa* treatment did not significantly alter FRAP and GSH levels (Fig 3B and 3D, *p >* 0.05) but did significantly decrease sulfhydryl levels (Fig 3F, *p* < 0.05).

### Effect of *V*. *rufa* extract on antioxidant enzyme activity in the pancreas

Effects of *V*. *rufa* extract on SOD, CAT, GPx, and GST activities are shown in (Fig 3I–3P). In diabetic-control rats, we observed increased SOD (Fig 3J, *p* < 0.05), CAT (Fig 3L, *p* < 0.05), GPx (Fig 3N, *p* < 0.05), and GST (Fig 3P, *p* < 0.05) activities. Diabetic-*V*. *rufa* treatment did not alter SOD, CAT, and GST (Fig 3J, 3L and 3P) activities but significantly decreased GPx (*p* < 0.05) activity.

### No effect of *V*. *rufa* extract on insulin-positive cells

**Fig 4:**
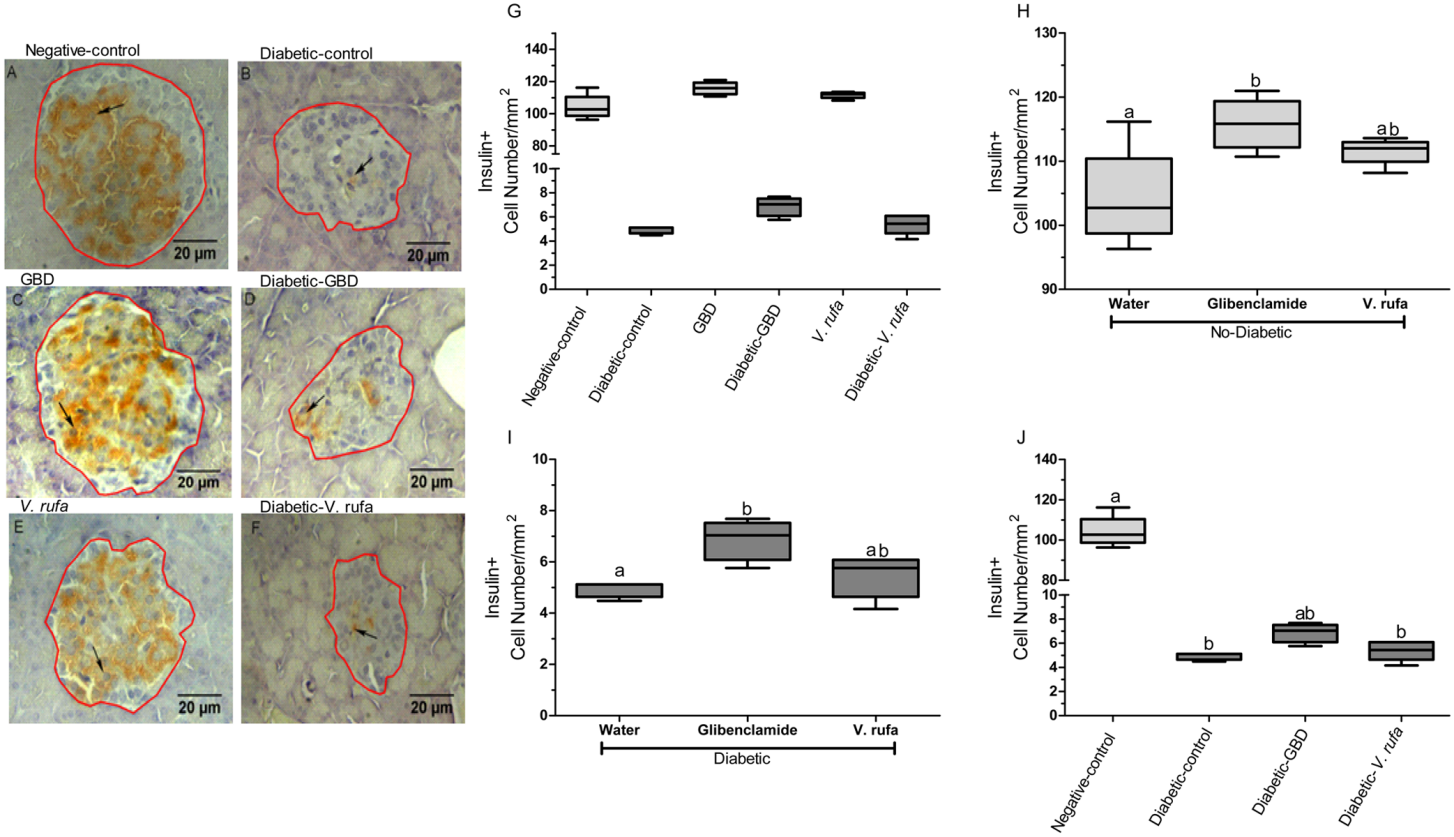
Evaluation of the number of insulin-positive β-cells in experimental groups. Rats were divided into negative-control and diabetic-control groups and were then treated with *V*. *rufa* extract (500 mg/kg) or glibenclamide (6 mg/kg). The presence of insulin-positive β-cells was analyzed in the islets of Langerhans in 5-μm cuts stained with rabbit anti-insulin (n = 5 animals per group, two independent replicates). (A–F) Immunohistochemical images of insulin-positive cells. Areas marked in red delimit islets, and arrows point to positive cells (brown). (G) Quantification of insulin-positive cells per mm^2^ in all experimental groups. (H, I) Comparison of number of insulin-positive cells in nondiabetic and diabetic groups, respectively. (J) Comparison of number of insulin-positive cells in diabetics vs. nondiabetics. Lowercase letters indicate statistically significant differences between groups (*p* < 0.05).

Results of the immunohistochemical analysis are shown in Fig 4. STZ administration caused a decrease in the number of cells positive for insulin. Diabetic-GBD rats exhibited an increased number of cells positive for insulin. Rats treated with *V*. *rufa* did not exhibit significant alterations in the number of insulin-positive pancreatic β-cells.

## Discussion

In the state of Minas Gerais in Brazil, *V*. *rufa* has been used for glycemic control by patients with type 2 diabetes mellitus. A doctor working in a primary health care unit reported that a patient with type 1 diabetes mellitus reduced their insulin use by 50% after treatment with an aqueous extract of macerated *V*. *rufa* stem bark. The present study reports for the first time the hypoglycemic, antihyperglycemic, and antioxidant effects of *V*. *rufa* in an experimental animal model of induced diabetes, thus scientifically validating this traditional claim.

In this study, we verified that an extract from the stem bark of *V*. *rufa* contains sugar. Consistent with this result, it was demonstrated that *Coptis chinensis* contains 96.3% carbohydrate, 4.8% uronic acid, and 0.61% protein and is mainly composed of glucose, arabinose, xylose, galactose, and galacturonic acid [27]. This is in contrast to other studies, which have described the presence of triterpenes in *Vochysia* species, including *V*. *divergens* [28], *Vochysia ferruginea* [29], *Vochysia pacifica* [30], and *V*. *tucanorum* [31].

A single oral administration of 5,000 mg/kg of *V*. *rufa* did not produce any signs of acute toxicity or mortality in the animals studied. Similar results have been reported by Gomes et al. [31], who administered a methanolic leaf extract of *V*. *tucanorum*, and by Hayes [32], with doses of up to 5,000 mg/kg considered non-toxic. These data indicate the permissibility of further pharmacological studies with this extract.

We found that untreated diabetic rats exhibited higher blood glucose levels and lower body weights than nondiabetic controls. Under diabetic conditions, the postprandial blood glucose level is not controlled efficiently due to insufficient insulin secretion [33]. Interestingly, the diabetic-*V*. *rufa* group exhibited significantly lower glucose levels compared with those of the diabetic-control group. However, control of blood glucose levels after 43 days of treatment was not supported by immunohistochemical analysis of the pancreas. Thus, *V*. *rufa* may not decrease blood glucose by increasing pancreatic secretion of insulin from existing β-cells.

Diabetic rats demonstrated elevated serum concentrations of urea, AST, ALT, ALP, and HDL-C, with a reduction in LDL levels when compared to those in nondiabetic rats. Elevated serum urea and creatinine levels are significant markers of renal dysfunction in diabetic hyperglycemia [34]. Activities of serum enzymes, including ALP and ALT, are used to evaluate hepatic disorders, with increases in the activities of these enzymes reflecting active liver damage [35, 36] While the *V*. *rufa* extract decreased glycemic levels, it did not alter AST, ALT, or ALP levels in diabetic rats. STZ produces an increase in ROS, causing lipid peroxidation in the membranes of adipose tissue. From this perspective, STZ increases lipid peroxidation and, consequently, hepatic enzyme release. While administration of *V*. *rufa* extract reduced glycemic levels, it did not alter liver enzymes or lipid peroxidation in diabetic rats. In nondiabetic rats, peroxidation and AST levels increased. Treatment with *V*. *rufa* extract also did not affect total cholesterol or triglyceride levels, suggesting that the extract did not affect lipid metabolism. Pandikumar et al. have reported similar results in diabetic rats [37].

In addition to the metabolic alterations caused by diabetes, chronic hyperglycemia promotes endogenous free radical generation and depletes antioxidant defense systems. Pancreatic damage is also an important stage in the development of diabetes and its complications. Pancreatic β-cells are highly prone to oxidative stress and damage because they express low levels of antioxidant enzymes and low antioxidant enzyme activity; these enzymes are the first line of defense against oxidative stress [38]. We observed an increase in CAT, GPx, and GST activities, in addition to increased TBARS and sulfhydryl levels and reduced GSH levels in diabetic rats compared with those in nondiabetic rats. Similar results have been reported in other studies of diabetic rat pancreata, including increases in CAT, GPx, and GST activities [5, 39, 40]. Consistent with our findings, Cumaoglu et al. found that the flavonoid fluvastatin did not alter SOD activity in the pancreata of diabetic rats [39]. An increase in GPx activity in the diabetic rat pancreas may represent a compensatory mechanism for detoxifying organic and inorganic peroxides, including excess hydrogen peroxide generated by increased SOD activity [41, 42]. GPx has been reported to have a broader protective spectrum than CAT because, in addition to H_2_O_2_, GPx also metabolizes other hydroperoxides, including lipid hydroperoxides [43]. The accumulation of H_2_O_2_ and other hydroperoxides may induce GPx activity, leading to its upregulation in diabetic rats. The reduced expression of GPx observed in rodent and human islets suggests that a typical approach for protection of β-cells against oxidative stress likely involves overexpression of GPx [44]. Thus, the overexpression of GPx could be a protective mechanism against oxidative stress. Our results indicate that the administration of *V*. *rufa* extract significantly decreases GPx activity and sulfhydryl levels in diabetic rats. Similarly, glibenclamide was found to reduce CAT, GPx, and GST activities and TBARS levels in diabetic rats. In the *V*. *rufa* group, FRAP levels increased, as was observed in the GBD group. The catalytic activity of GPx is complementary to that of catalase; the *k*_m_ of GPx (*k*_m_ = 0.25 mmol/L) is lower than that of catalase (*k*_m_ = 25 mmol/L), providing a preferential pathway for the degradation of low concentrations of H_2_O_2_ in intact cells [45]. Therefore, treatment with *V*. *rufa* extract had no effect on catalase activity.

Consistent with our findings, Ardestani et al. observed a decrease in GSH levels in the pancreata of diabetic rats compared to levels in nondiabetic rats [46] GSH is a major intracellular redox buffer that participates in cellular defense against oxidative stress by scavenging free radicals and reactive oxygen intermediates [47]. Thus, a decrease in pancreatic GSH levels in diabetic rats may reflect a direct reaction between GSH and the free radicals generated by hyperglycemia in diabetes mellitus.

The elevated pancreatic lipid peroxidation observed in the diabetic rats can be attributed to enhanced production of ROS, which leads to oxidative stress [48]. The products of lipid peroxidation typically oxidize protein sulfhydryl groups, resulting in the formation of disulfide bonds. Thus, increased levels of sulfhydryl groups in diabetic rats are related to an increase in TBARS levels. While the extract of *V*. *rufa* reduced sulfhydryl levels, a reduction in TBARS levels was not observed in the pancreas.

In this study, we demonstrated the presence of sugars in the *V*. *rufa* extract that may be responsible for scavenging free radicals released by STZ and thus enhancing both enzymatic and non-enzymatic antioxidants in treated diabetic rats. Previous studies have also demonstrated that polysaccharides from other sources can increase antioxidant enzyme activities in the blood and tissues of diabetic mice or rats [6, 7, 9, 49–52]. Moreover, a recent study reported that the hypoglycemic mechanisms of polysaccharides are closely associated with their antioxidant activities [27]. Based on these findings, our study suggests that one mechanism of the antihyperglycemic action of *V*. *rufa* extract may be tied to its antioxidant activity and free radical scavenging ability, which provide protection against oxidative damage. Zhang et al. (2012) [52] demonstrated that orally administered *Taxus cuspidata* polysaccharides had a hypoglycemic effect and could alleviate oxidative stress in the kidney and liver of STZ-induced diabetic rats. Moraes et al. (2015) [15] also showed that the reducing sugars in *V*. *rufa* extract efficiently reduced hepatic oxidative stress caused by STZ-induced diabetes and produced no morphological changes according to histological analysis.

In conclusion, this is the first report of the antioxidant and antidiabetic effects of an aqueous extract of the *V*. *rufa* stem bark. Our results indicate that the sugars of the extract are capable of reducing blood glucose levels and alleviating oxidative stress parameters in the pancreatic tissue of STZ-induced diabetic rats. This protective effect against oxidative stress in the pancreas may also partially contribute to the antihyperglycemic effect of *V*. *rufa* extract in diabetic rats. *V*. *rufa* extract therefore represents a potential candidate for pharmaceutical evaluation pending full structural characterization of the active compounds possessing antidiabetic and antioxidative activities.

## References

[1] Danaei G, Finucane MM, Lu Y, Singh GM, Cowan MJ, Paciorek CJ, et al National, regional, and global trends in fasting plasma glucose and diabetes prevalence since 1980: systematic analysis of health examination surveys and epidemiological studies with 370 country-years and 2.7 million participants. Lancet. 2011;378: 31–40. doi: 10.1016/S0140-6736(11)60679-X 21705069 10.1016/S0140-6736(11)60679-X

[2] Hunt JV, Smith CC, Wolff SP. Autoxidative glycosylation and possible involvement of peroxides and free radicals in LDL modification by glucose. Diabetes. 1990;39: 1420–1424. 2227114 10.2337/diab.39.11.1420

[3] Robertson RP. Chronic oxidative stress as a central mechanism for glucose toxicity in pancreatic islet beta cells in diabetes. J Biol Chem. 2004;279: 42351–42354. doi: 10.1074/jbc.R400019200 15258147 10.1074/jbc.R400019200

[4] Saxena AK, Srivastava P, Kale RK, Baquer NZ. Impaired antioxidant status in diabetic rat liver. Effect of vanadate. Biochem Pharmacol. 1993;45: 539–542. 8442752 10.1016/0006-2952(93)90124-f

[5] Kakkar R, Kalra J, Mantha SV, Prasad K. Lipid peroxidation and activity of antioxidant enzymes in diabetic rats. Mol Cell Biochem. 1995;151: 113–119. 8569756 10.1007/BF01322333

[6] Chen X, Tang J, Xie W, Wang J, Jin J, Ren J, et al Protective effect of the polysaccharide from *Ophiopogon japonicus* on streptozotocin-induced diabetic rats. Carbohydr Polym. 2013;94: 378–385. doi: 10.1016/j.carbpol.2013.01.037 23544552 10.1016/j.carbpol.2013.01.037

[7] Li XM. Protective effect of *Lycium barbarum* polysaccharides on streptozotocin-induced oxidative stress in rats. Int J Biol Macromol. 2007;40: 461–465. doi: 10.1016/j.ijbiomac.2006.11.002 17166579 10.1016/j.ijbiomac.2006.11.002

[8] Zou S, Zhang X, Yao W, Niu Y, Gao X. Structure characterization and hypoglycemic activity of a polysaccharide isolated from the fruit of *Lycium barbarum* L. Carbohydr Polym. 2010;80: 1161–1167.

[9] Zhao LY, Lan QJ, Huang ZC, Ouyang LJ, Zeng FH. Antidiabetic effect of a newly identified component of *Opuntia dillenii* polysaccharides. Phytomedicine. 2011;18: 661–668. doi: 10.1016/j.phymed.2011.01.001 21300531 10.1016/j.phymed.2011.01.001

[10] Yazdanparast R, Ardestani A, Jamshidi S. Experimental diabetes treated with *Achillea santolina*: effect on pancreatic oxidative parameters. J Ethnopharmacol. 2007;112: 13–18. doi: 10.1016/j.jep.2007.01.030 17336007 10.1016/j.jep.2007.01.030

[11] Schultes RE, Raffauf RF. The healing forest: Medicinal and toxic plants of the northwest Amazonia. Portland: Dioscorides Press; 1990.

[12] Gomes Rde C, Bonamin F, Darin DD, Seito LN, Di Stasi LC, Dokkedal AL, et al Antioxidative action of methanolic extract and buthanolic fraction of *Vochysia tucanorum* Mart. in the gastroprotection. J Ethnopharmacol. 2009;121: 466–471. doi: 10.1016/j.jep.2008.11.013 19071206 10.1016/j.jep.2008.11.013

[13] Hess SC, Brum RL, Honda NK, Cruz AB, Moretto E, Cruz RB, et al Antibacterial activity and phytochemical analysis of *Vochysia divergens* (Vochysiaceae). J Ethnopharmacol. 1995;47: 97–100. 7500642 10.1016/0378-8741(95)01260-k

[14] Silva MAB, Melo LVL, Ribeiro RV, Souza JPM, Lima JCS, Martins DTO, et al Ethnobotanical survey of plants used as anti-hyperlipidemic and anorexigenic by the population of Nova Xavantina-MT, Brazil. Rev Bras Farmacogn. 2010;20: 549–562.

[15] Moraes IB, Manzan-Martins C, Gouveia NM, Calabria LK, Hiraki KRN, Moraes AS, et al Polyploidy analysis and attenuation of oxidative stress in hepatic tissue of STZ-induced diabetic rats treated with an aqueous extract of *Vochysia rufa*. Evid Based Complement Alternat Med. 2015;2015: 1–8. 10.1155/2015/316017 PMC4339860 25763088

[16] Calábria LK, Costa AV, Oliveira RJS, Deconte SR, Nascimento R, Carvalho WJ, et al Myosins are differentially expressed under oxidative stress in chronic streptozotocin-induced diabetic rat brains. ISRN Neurosci. 2013;2013: 1–10. 10.1155/2013/423931 PMC4045535 24982856

[17] de Gouveia NM, Alves FV, Furtado FB, Scherer DL, Mundim AV, Espindola FS. An in vitro and in vivo study of the α-amylase activity of phaseolamin. J Med Food. 2014;17: 915–20. doi: 10.1089/jmf.2013.0044 24650210 10.1089/jmf.2013.0044 PMC4126268

[18] Bradford MM. A rapid and sensitive method for the quantitation of microgram quantities of protein utilizing the principle of protein-dye binding. Anal Biochem. 1976;72: 248–254. 942051 10.1016/0003-2697(76)90527-3

[19] Benzie IF, Strain JJ. Ferric reducing/antioxidant power assay: direct measure of total antioxidant activity of biological fluids and modified version for simultaneous measurement of total antioxidant power and ascorbic acid concentration. Methods Enzymol. 1999;299: 15–27. 9916193 10.1016/s0076-6879(99)99005-5

[20] Hermes-Lima M, Willmore WG, Storey KB. Quantification of lipid peroxidation in tissue extracts based on Fe(III)xylenol orange complex formation. Free Radic Biol Med. 1995;19: 271–280. 7557541 10.1016/0891-5849(95)00020-x

[21] Faure P, Lafond JL. Measurement of plasma sulfhydryl and carbonyl groups as a possible indicator of protein oxidation In: Favier AE, Cadet J, Kalnyanaraman M, Fontecave M, Pierre JL, editors. Analysis of free radicals in biological systems. Basel: Birkhaüser; 1995 pp. 237–249.

[22] Habig WH, Pabst MJ, Jakoby WB. Glutathione S-transferases. The first enzymatic step in mercapturic acid formation. J Biol Chem. 1974;249: 7130–7139. 4436300

[23] Aebi H, Suter H, Feinstein RN. Activity and stability of catalase in blood and tissues of normal and acatalasemic mice. Biochem Genet. 1968;2: 245–251. 5715189 10.1007/BF01474764

[24] Misra HP, Fridovich I. The role of superoxide anion in the autoxidation of epinephrine and a simple assay for superoxide dismutase. J Biol Chem. 1972;247: 3170–3175. 4623845

[25] Flohe L, Gunzler WA. Assays of glutathione peroxidase. Methods Enzymol. 1984;105: 114–121. 6727659 10.1016/s0076-6879(84)05015-1

[26] Beutler E, Duron O, Kelly BM. Improved method for the determination of blood glutathione. J Lab Clin Med. 1963;61: 882–888. 13967893

[27] Jiang S, Du P, An L, Yuan G, Sun Z. Anti-diabetic effect of *Coptis chinensis* polysaccharide in high-fat diet with STZ-induced diabetic mice. Int J Biol Macromolec. 2013;55: 118–122. 10.1016/j.ijbiomac.2012.12.035 23295205

[28] Hess SC, Brum RL, Honda NK, Cruz AB, Moretto E, Cruz RB, et al Antibacterial activity and phytochemical analysis of *Vochysia divergens* (Vochysiaceae). J Ethnopharmacol. 1995;47: 97–100. 7500642 10.1016/0378-8741(95)01260-k

[29] Zucaro YL, Compagnonea RS, Hess SC. β-hydroxymaslinic acid, a triterpene from *Vochysia ferruginea*. J Braz Chem Soc. 2000;11: 241–244.

[30] Weniger B, Lobstein A, Um BH, Vonthron-Senechau C, Anton R, Usuga NJ, et al Bioactive triterpenoids from *Vochysia pacifica* interact with cyclic nucleotide phosphodiesterase isozyme PDE4. Phytother Res. 2005;19: 75–77. doi: 10.1002/ptr.1613 15798995 10.1002/ptr.1613

[31] Gomes R de C, Bonamin F, Darin DD, Seito LN, Di Stasi LC, Dokkedal AL, et al Antioxidative action of methanolic extract and buthanolic fraction of *Vochysia tucanorum* Mart. in the gastroprotection. J Ethnopharmacol. 2009;121: 466–471. doi: 10.1016/j.jep.2008.11.013 19071206 10.1016/j.jep.2008.11.013

[32] Loomis TA, Hayes AW. Essentials of Toxicology. 4th ed London: Academic Press Limited; 1996.

[33] Wu C, Li Y, Chen Y, Lao X, Sheng L, Dai R, et al Hypoglycemic effect of *Belamcanda chinensis* leaf extract in normal and STZ-induced diabetic rats and its potential active faction. Phytomedicine. 2010;18: 292–297. doi: 10.1016/j.phymed.2010.07.005 20739161 10.1016/j.phymed.2010.07.005

[34] Almdal TP, Vilstrup H. Strict insulin therapy normalises organ nitrogen contents and the capacity of urea nitrogen synthesis in experimental diabetes in rats. Diabetologia. 1988;31: 114–118. 3282951 10.1007/BF00395558

[35] Fortson WC, Tedesco FJ, Starnes EC, Shaw CT. Marked elevation of serum transaminase activity associated with extrahepatic biliary tract disease. J Clin Gastroenterol. 1985;7: 502–505. 4086745 10.1097/00004836-198512000-00012

[36] Hultcrantz R, Glaumann H, Lindberg G, Nilsson LH. Liver investigation in 149 asymptomatic patients with moderately elevated activities of serum aminotransferases. Scand J Gastroenterol. 1986;21: 109–113. 3952445 10.3109/00365528609034632

[37] Pandikumar P, Babu NP, Ignacimuthu S. Hypoglycemic and antihyperglycemic effect of *Begonia malabarica* Lam. in normal and streptozotocin induced diabetic rats. J Ethnopharmacol. 2009;124: 111–115. doi: 10.1016/j.jep.2009.04.001 19443148 10.1016/j.jep.2009.04.001

[38] Lenzen S. Oxidative stress: the vulnerable beta-cell. Biochem Soc Trans. 2008;36: 343–347. doi: 10.1042/BST0360343 18481954 10.1042/BST0360343

[39] Cumaoglu A, Ozansoy G, Irat AM, Aricioglu A, Karasu C, Ari N. Effect of long term, non cholesterol lowering dose of fluvastatin treatment on oxidative stress in brain and peripheral tissues of streptozotocin-diabetic rats. Eur J Pharmacol. 2010; 654: 80–85. doi: 10.1016/j.ejphar.2010.11.035 21172345 10.1016/j.ejphar.2010.11.035

[40] Erejuwa OO, Sulaiman SA, Wahab MS, Sirajudeen KN, Salleh MS, Gurtu S. Antioxidant protection of Malaysian tualang honey in pancreas of normal and streptozotocin-induced diabetic rats. Ann Endocrinol. 2010;71: 291–296. 10.1016/j.ando.2010.03.003 20398890

[41] Christophersen BO. Reduction of linolenic acid hydroperoxide by a glutathione peroxidase. Biochim Biophys Acta. 1969;176: 4463–70. 10.1016/0005-2760(69)90213-6 5800037

[42] Dobrina A, Patriarca P. Neutrophil-endothelial cell interaction. Evidence for and mechanisms of the self-protection of bovine microvascular endothelial cells from hydrogen peroxide-induced oxidative stress. J Clin Invest. 1986;78: 462–471. doi: 10.1172/JCI112598 3734101 10.1172/JCI112598 PMC423583

[43] Christophersen BO. Formation of monohydroxy-polyenic fatty acids from lipid peroxides by a glutathione peroxidase. Biochim Biophys Acta. 1968;164: 35–46. 5680294 10.1016/0005-2760(68)90068-4

[44] Robertson RP, Harmon JS. Pancreatic islet beta-cell and oxidative stress: the importance of glutathione peroxidase. FEBS Lett. 2007;581: 3743–3748. doi: 10.1016/j.febslet.2007.03.087 17433304 10.1016/j.febslet.2007.03.087 PMC2762945

[45] Ugochukwu NH, Cobourne MK. Modification of renal oxidative stress and lipid peroxidation in streptozotocin-induced diabetic rats treated with extracts from *Gongronema latifolium* leaves. Clin Chim Acta. 2003;336: 73–81. 14500037 10.1016/s0009-8981(03)00325-5

[46] Ardestani A, Yazdanparast R, Jamshidi S. Therapeutic effects of *Teucrium polium* extract on oxidative stress in pancreas of streptozotocin-induced diabetic rats. J Med Food. 2008;11: 525–532. doi: 10.1089/jmf.2006.0230 18800902 10.1089/jmf.2006.0230

[47] Wu G, Fang YZ, Yang S, Lupton JR, Turner ND. Glutathione metabolism and its implications for health. J Nutr. 2004;134: 489–492. 14988435 10.1093/jn/134.3.489

[48] Ilhan N, Halifeoglu I, Ozercan HI. Tissue malondialdehyde and adenosine triphosphatase level after experimental liver ischaemia-reperfusion damage. Cell Biochem Funct. 2001;19: 207–212. doi: 10.1002/cbf.912 11494310 10.1002/cbf.912

[49] Xue SX, Chen XM, Lu JX, Jin LQ. Protective effect of sulfated *Achyranthes bidentata* polysaccharides on streptozotocin-induced oxidative stress in rats. Carbohydr Polym. 2009;75: 415–419.

[50] Jia J, Zhang X, Hu Y-S, Wu Y, Wang Q-Z, Li N-N, et al Evaluation of in vivo antioxidant activities of *Ganoderma lucidum* polysaccharides in STZ-diabetic rats. Food Chem. 2009;115: 32–36.

[51] Liu YT, Sun J, Rao SQ, Su YJ, Yang YJ. Antihyperglycemic, antihyperlipidemic and antioxidant activities of polysaccharides from *Catathelasma ventricosum* in streptozotocin-induced diabetic mice. Food Chem Toxicol. 2013;57: 39–45. doi: 10.1016/j.fct.2013.03.001 23500773 10.1016/j.fct.2013.03.001

[52] Zhang D, Meng H, Yang H-S. Antidiabetic activity of *Taxus cuspidata* polysaccharides in streptozotocin-induced diabetic mice. Int J Biol Macromolec. 2012;50: 720–724. 10.1016/j.ijbiomac.2011.12.020 22214824

